# Insight into the surface activity of defect structure in α-MnO_2_ nanorod: first-principles research

**DOI:** 10.1038/s41598-021-83861-2

**Published:** 2021-02-26

**Authors:** Pengsen Zhao, Guifa Li, Haizhong Zheng, Shiqiang Lu, Ping Peng

**Affiliations:** 1 School of Material Science and Engineering, Nanchang Hangkong University, Jiangxi, 330063 China; 2 School of Material Science and Engineering, Hunan University, Hunan, 410082 China

**Keywords:** Mathematics and computing, Nanoscale materials, Computational nanotechnology

## Abstract

The contribution of defect structure to the catalytic property of α-MnO_2_ nanorod still keeps mysterious right now. Using microfacet models representing defect structure and bulk models with high Miller index, several parameters, such as cohesive energy, surface energy, density of state, electrostatic potential, et al*.*, have been used to investigate the internal mechanism of their chemical activities by first-principles calculation. The results show that the trend in surface energies of microfacet models follows as *E*_surface_[(112 × 211)] > *E*_surface_[(110 × 211)] > *E*_surface_[(100 × 211)] > *E*_surface_[(111 × 211)] > *E*_surface_[(112 × 112)] > *E*_surface_[(111 × 112)], wherein all of them are larger than that of bulk models. So the chemical activity of defect structure is much more powerful than that of bulk surface. Deep researches on electronic structure show that the excellent chemical activity of microfacet structure has larger value in dipole moments and electrostatic potential than that of bulk surface layer. And the microfacet models possess much more peaks of valent electrons in deformantion electronic density and molecular orbital. Density of state indicates that the excellent chemical activity of defect structure comes from their proper hybridization in *p* and *d* orbitals.

## Introduction

Environmental contamination such as heavy metallic ions in water, volatile organic compounds, poses a serious threat to human health and safety^1–3^. Manganese dioxide (MnO_2_), which possesses high natural content, safety, environmental friendliness, low cost, good physical and chemical properties, has attracted great attention in catalyst and adsorbing application right now^4^. Thousands of MnO_2_ nanomaterials have been produced in the laboratory^5,6^, such as nanorods^7^, nanoparticles^8^, nanowires^9^, nanourchins^10^ and so on. Débart et al.^11^ has pointed out that the chemical activity of α-MnO_2_ nanowires is better than the corresponding bulk materials. Thus all of experimental researches hope to get optimal catalyst performance of α-MnO_2_ by nanotechnology^11^. Much more activity sites are the common consensus for the excellent performance of nanomaterials. But restricted by the lowest energy rule, all of the α-MnO_2_ nanomaterials and bulk materials have the same basic Miller index as {110}, {200} and {211}, {310}^9,12^. Luo et al.^12^ investigated the removal mechanism of As and Sb ions on α-MnO_2_ nanorod through experimental and theoretical method. Based on (100) and (110) bulk surface, he revealed that the surface energy and valent electrons of surface layer in α-MnO_2_ nanorod determined the removal ability of As and Sb ions. However, Tompsett et al*.*^13^ illuminated that the geometric morphology of α-MnO_2_ nanorod was composed with serial low Miller index (100) and (110) and high Miller index (211) and (111) bulk surface, wherein the surface activity of (211) and (111) bulk surface could not be ignored. So Jia et al.^14^ studied the influence of α-MnO_2_ geometric morphology on its catalytic ozonolysis activity by (211), (110) and (200) bulk surface models, which showed that the (211) bulk surface model with largest specific surface area and the largest oxygen vacancy possessed the best chemical activity. As well known, all of their differences in chemical activity of α-MnO_2_ nanomaterials originate from their activity sites not only in surface layer but also in defect structure^8^. Surface deficiencies sites, which are regarded as an effective way to tune catalytic reaction kinetics, activation energetics and reactive mechanism, produce the main activity sites by releasing much more vigor from valent electrons^15^. Kubo et al*.*^16^ investigated the properties of rutile TiO_2_ changed along with the roughness of surface layer by noncontact atomic force microscopy (NC-AFM) and density functional calculations. Through microfacet models, he found that the surface stability and geometric morphology restructuring were mainly influenced by density of dangling bonds. Based on microfacet models, Ogawa et al*.*^17^ also researched the adsorbing ability and chemical catalytic of oxygen atom on Pt roughness surface with defect structure. Compared with Pt(211), (111) and (100), it was found that the activity site was placed on the pyramid structure of Pt(211) surface layer. Zhou et al*.*^18^ discussed the preferential corrosion sites of YSZ (Yttria-stabilized zirconia) columnar crystal affected by CMAS (CaO–MgO–Al_2_O_3_–SiO_2_) melt through two different models as bulk models representing surface layer and microfacet models representing corner structure. It was found that the vigorous chemical activity of corner structure was unfavorable to the corrosion resistance of YSZ columnar crystal. In short the key to open the chemical property of nanomaterials is to scan and pry the activity sites. α-MnO_2_ nanomaterials exhibit excellent chemical activity^13^, but their spring of chemical activity still confuse and attract many researchers' attention. In this paper, electronic properties of surface layer in α-MnO_2_ nanomaterials are studied systematically by high Miller index bulk surface models and microfacet models representing defect structure.

## Simulation models and method

**Figure 1:**
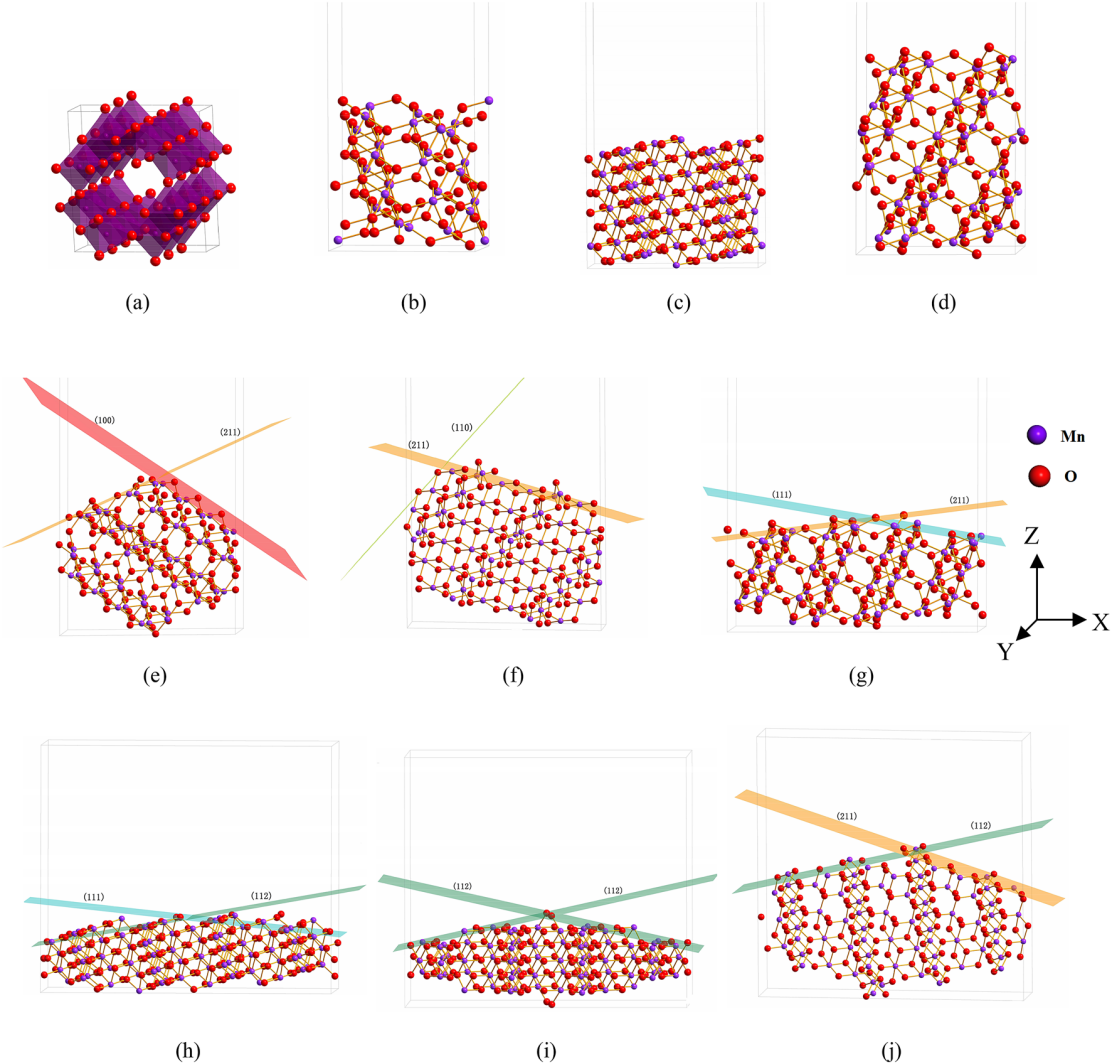
Several simulated α-MnO_2_ bulk surface and microfacet models, wherein (**a**) crystal (Mn_8_O_16_), (**b**) (111) surface (Mn_32_O_64_), (**c**) (112) surface (Mn_56_O_112_), (**d**) (211) surface (Mn_32_O_64_), (**e**) [(100 × 211)] microfacet (Mn_60_O_120_), (**f**) [(110 × 211)] microfacet (Mn_56_O_112_), (**g**) [(111 × 211)] microfacet (Mn_54_O_108_), (**h**) [(111 × 112)] microfacet (Mn_76_O_152_), (**i**) [(112 × 112)] microfacet (Mn_68_O_136_), (**j**) [(112 × 211)] microfacet (Mn_72_O_144_).

According with former experimental research^19^ and theoretical nanorod model constructed by Wulff method^13^, several defect structures modeled by microfacet and bulk surface with high Miller index were constructed and simulated systematically as shown in Figs. S1 and 1. In previous paper^20^, the chemical activity of surface layer with low Miller index as (100) and (110) bulk surface has been exposed detailed absolutely. In this paper every model also contains a vacuum thickness not less than 10 Ǻ to separate their interactions between two slabs^21^. According with previous research^20^, the lattice parameters of α-MnO_2_ bulk unit cell are equal to *a* = *b* = 9.922 Å and *c* = 2.904 Å in Fig. 1a. Several α-MnO_2_ bulk surface models with high Miller index as (111), (112) and (211) are constructed in Fig. 1b–d. The corresponding defect structures representing by microfacet models as [(100 × 211)], [(110 × 211)], [(111 × 211)], [(111 × 112)], [(112 × 112)] and [(112 × 211)] are constructed via the above different Miller index planes in Fig. 1e–j, wherein [(100 × 211)] and [(110 × 211)] microfacet models also refer to some low Miller index (100) and (110) bulk surface models constructed by Chen et al.^22^. All of the simulated models in this paper were relaxed by density functional theory (DFT) embedded in Cambridge Sequential Total Energy Package (CASTEP) code with plane waves and pseudopotentials^23^. Then their electronic structure was calculated using the Generalized Gradient Approximation (GGA) of Perdew, Burke, and Ernzerh with Hubbard U correction^24^. A minimum of 8 × 1 × 1 k-points were used in the Brillouin zone of the conventional cell and scaled appropriately for supercells. The cutoff energy in the bulk models are equal to 450 eV and that of microfacet models equal to 400 eV. To further improve the calculation accuracy of α-MnO_2_ surface, the field coulomb potential correction for the 3*d* orbital electronic structure of Mn atoms was carried out^12^. All calculations were performed in a ferromagnetic orders spin polarized configuration, while effects of more complex magnetic orders were left for future work due to their low energy scale. The geometric optimization of electronic configuration with Hubbard U = 1.6 eV suggested by previous paper^25^. The calculated lattice parameters for α-MnO_2_ obtained from PBE + U are within 1.8% of the theoretical^13,26^ and experimental^27,28^ parameters as shown in Supplementary Table S1. All of the atomic positions in these primitive cells were relaxed according to the total energy and force using the BFGS scheme^29^, based on the cell optimization criteria (RMS force of 0.1 eV/Å, a stress of 0.2 GPa, and displacement of 0.005 Å). The convergence criteria of self-consistent field (SCF) and energy tolerances were set at 1.0 × 10^–4^ and 5.0 × 10^–4^ eV/atom, respectively.

## Results and discussion

### Structural stability and surface activity

**Figure 2:**
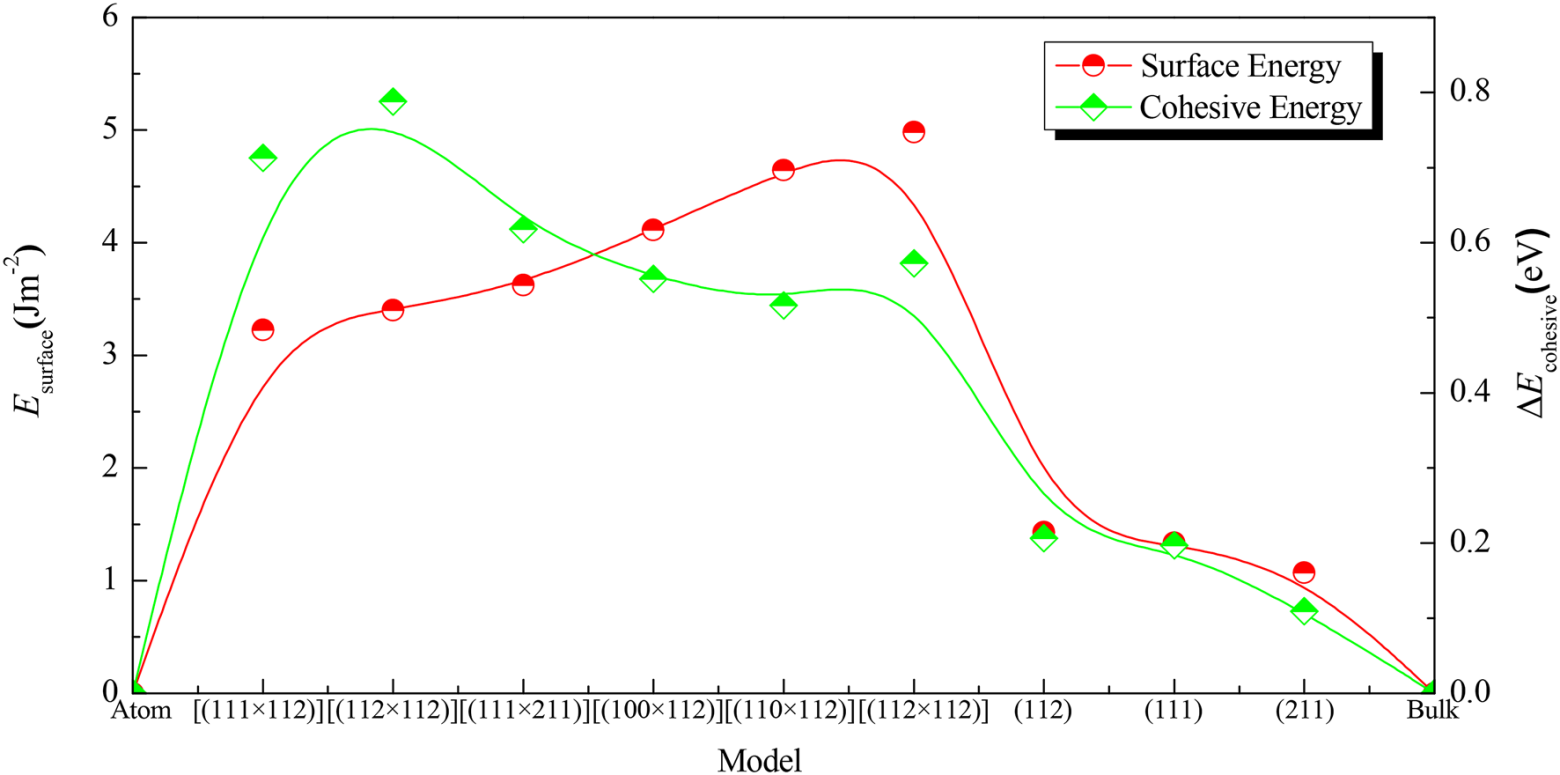
Schematic diagram of the surface energy and cohesive energy for MnO_2_ bulk surface and microfacet.

Surface energy (*E*_surface_) and cohesive energy (*E*_cohesive_) are used to evaluate the structure stability and surface activity of the α-MnO_2_ crystal, bulk surfaces and microfacets models. From definition, the surface energy is calculated by taking the difference between the energy of a constructed slab and the same number of α-MnO_2_ formula units. Cohesive energy is representative of the work required for a crystal to be decomposed into single atoms. They are calculated by following Eqs. (1) and (2), respectively:

1 $$ E_{{{\text{surface}}}} { = }\frac{{E_{{{\text{total}}}} - nE_{{\text{b}}} }}{2 \cdot S} $$

2 $$ E_{{{\text{cohesive}}}} = \frac{1}{l + m}\left( {E_{{{\text{total}}}}^{{{\text{Mn}}_{l} {\text{O}}_{m} }} - lE_{{{\text{gas}}}}^{{{\text{Mn}}}} - mE_{{{\text{gas}}}}^{{\text{O}}} } \right) $$

wherein *E*_total_ represents the total energy of bulk surface or microfacet models. *E*_b_ represents the total energy of crystal. *n* represents the number of basic units composed bulk surface or microfacets. *S* represents the same area belonging to the upper or lower surface. *l* and *m* are the number of Mn and O atoms in every surface models respectively. $E_{{{\text{total}}}}^{{{\text{Mn}}_{l} {\text{O}}_{m} }}$ is the total energy of Mn_*l*_O_*n*_ surface models. *E*Mn gas and *E*O gas are the energies of Mn and O atoms in the gas state, respectively. For getting Mn and O gaseous atoms, a 10 Å × 10 Å × 10 Å box was built with a single atom in the center, wherein *E*Mn gas = -588.1855 eV and *E*O gas = -432.2548 eV, respectively^22^. The results are shown in Table 1 and Fig. 2.

**Table 1:** Surface energy (*E*_surface_) and cohesive energy (*E*_cohesive_) of MnO_2_ crystal, bulk surface and microfacet models.

	Models	Miller index	*K* points	*E*_total_ (ev)	*a* (Å)	*b* (Å)	*s*(Å^2^)	*E*_surface_ (Jm^−2^)	*E*_cohesive_ (eV)
Crystal	Mn_8_O_16_	Mn_8_O_16_	8 × 1 × 1	− 11,734.8986				–	− 4.7223
	Mn_32_O_64_	(111)	1 × 5 × 1	− 43,988.1132	10.3382	10.3382	106.5460	1.3333, 1.32^13^	− 4.5251
Bulk surface	Mn_56_O_112_	(112)	1 × 1 × 1	− 85,042.0537	14.0318	14.3291	201.0645	1.4308, 1.40^13^	− 4.5157
	Mn_32_O_64_	(211)	1 × 1 × 1	− 70,393.6579	11.4969	10.3382	117.6548	1.0698, 1.08^13^	− 4.6131
Microfacet	Mn_60_O_120_	[(100 × 211)]	1 × 1 × 1	− 87,917.7053	18.5950	10.3382	182.8448	4.1143	− 4.1705
	Mn_56_O_112_	[(110 × 211)]	1 × 2 × 1	− 82,057.5144	21.0890	7.1646	149.4809	4.6441	− 4.2059
	Mn_54_O_108_	[(111 × 211)]	1 × 1 × 1	− 79,110.4855	21.5520	10.3382	221.0766	3.6216	− 4.1046
	Mn_76_O_152_	[(111 × 112)]	1 × 1 × 1	− 111,318.9420	14.0318	28.7360	403.2186	3.2259	− 4.0093
	Mn_68_O_136_	[(112 × 112)]	1 × 1 × 1	− 99,585.8633	14.0318	26.9440	378.0736	3.4020	− 3.9343
	Mn_72_O_144_	[(112 × 211)]	1 × 2 × 1	− 105,490.3518	27.7560	7.1646	198.6925	4.9820	− 4.1496

In our previous paper^22^, it is found that the *E*_surface_ values of (100) and (110) surfaces are similar to the results reported by Tompsett et al.^13^. Furthermore in Table 1 and Fig. 2, it is found that the surface energies of bulk surface in this paper, *i.e.*, *E*_surface_(112) (1.4308 Jm^−2^) > *E*_surface_(111) (1.3333 Jm^−2^) > *E*_surface_(211) (1.0698 Jm^−2^), wherein all of them are larger than that of *E*_surface_(110) = 0.75 Jm^−2^ and *E*_surface_(100) = 0.64 Jm^−2^^13^, are close to the results of Tompsett et al*.*^13^. This trend in surface energies is on the contrary with their cohesive energies, *i.e*., *E*_cohesive_(112) (-4.5157 eV) > *E*_cohesive_(111) (− 4.5251 eV) > *E*_cohesive_(211) (− 4.6131 eV). Then for bulk surface with high Miller index, the much less cohesive energy is, the much smaller surface energy is. Surface energy is an important parameter to estimate surface chemical activity. So it is hard to produce some bulk surface both possessing highest chemical activity and structural stability. For microfacet models representing defect structure in Fig. 1e–j, it is found the trend in surface energies is *E*_surface_[(112 × 211)] (4.9820 Jm^−2^) > *E*_surface_[(110 × 211)] (4.6441 Jm^−2^) > *E*_surface_[(100 × 211)] (4.1143 Jm^−2^) > *E*_surface_[(111 × 211)] (3.6216 Jm^−2^) > *E*_surface_[(112 × 112)] (3.4020 Jm^−2^) > *E*_surface_[(111 × 112)] (3.2259 Jm^−2^). However to their cohesive energies, the trend is *E*_cohesive_[(110 × 211)] (− 4.2059 eV) < *E*_cohesive_[(100 × 211)] (− 4.1705 eV) < *E*_cohesive_[(112 × 211)] (-4.1496 eV) < *E*_cohesive_[(111 × 211)] (− 4.1046 eV) < *E*_cohesive_[(111 × 112)] (− 4.0093 eV) < *E*_cohesive_[(112 × 112)] (− 3.9343 eV). From these trends an interesting phenomenon is extracted that the [(110 × 211)] microfacet has large surface energy, but its cohesive energy is the lowest, and the smallest surface energies of [(111 × 112)] and [(112 × 112)] microfacets have the largest cohesive energies. Comparing their surface energies and cohesive energies, it can be found that the surface energies of microfacet are significantly larger than that of the bulk models. So the surface chemical activity of defect structure modeled by microfacets is much more vigorous than that of bulk surface with high Miller index^30^. Then to MnO_2_ nanomaterials, the microfacet models have better representative in chemical activity and structural stability than the bulk surface models^22^.

Generally speaking, the microfacet models can be separated by two components of bulk surface, such as [(111 × 211)] microfacet is composed by (111) and (211) bulk surface as shown in Fig. 1. Deduced by intuitive thinking, they will have some relationship, especially to surface chemical activity. To our surprise, they have the inverse phenomenon. For (100), (110) and (211) bulk surface models, which have the smallest surface energies, but their composed microfacet models as [(100 × 211)] and [(110 × 211)] have the largest surface energies (*E*_surface_ = 4.1143 Jm^−2^ and *E*_surface_ = 4.6441 Jm^−2^). For (112) and (111) bulk surface models, which have the largest surface energies, but their composed microfacets as [(111 × 112)] and [(112 × 112)] have the smallest surface energies (*E*_surface_ = 3.2259 Jm^−2^ and *E*_surface_ = 3.4020 Jm^−2^) as shown in Table 1 and Fig. 2. Such phenomenon does not be reported by previous paper^13^. But it is very important for optimizing the nanostructure of bulk materials or microstructure of nanomaterials, which means in the process of manufacturing nanostructure it would not be the only way to aim at high Miller index surface. Systematically considering the trend of surface energy and cohesive energy (Fig. 2), there exists some microfacet in optimal structure with powerful surface chemical activity and structural stability, which is consist with the research of Tompsett et al.^13^. At last, some problems face us that what induce the contrary trend of surface energy and cohesive energy for bulk and nanomaterials?

### Density of state

**Figure 3:**
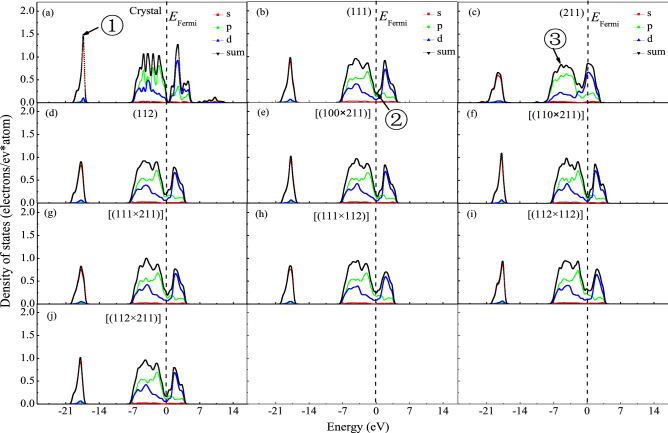
Partial density of states of MnO_2_ crystal, bulk surface and microfacet models.

The different trends in their structural stability and catalytic activity between bulk surface and microfacet with nanostructure come from their electronic structure along surface slab. Then their partial density of states (PDOS) per atom were calculated as shown in Fig. 3. From Fig. 3, it can be seen that the intensity of bonding peak at − 17.3 eV (labeled by ①) for crystal and bulk surface is different with each other, wherein PDOS_Crystal_ = 1.455 electrons/eV*atom > PDOS_(111)_ = 0.982 electrons/eV*atom > PDOS_(112)_ = 0.909 electrons/eV*atom > PDOS_(211)_ = 0.665 electrons/eV*atom. Then it is not hard to understand why the crystal has the minimum cohesive energy. Furthermore, along the boundary of Fermi facet (labeled by ②), the value of PDOS in crystal is very small, however those in (111), (112) and (211) bulk surface are large, wherein PDOSFermi crystal = 0.083 electrons/eV*atom < PDOSFermi (111) = 0.190 electrons/eV*atom < PDOSFermi (112) = 0.193 electrons/eV*atom < PDOSFermi (211) = 0.325 electrons/eV*atom. From definition, Fermi facet is the boundary of bonding region and antibonding region. The space between bonding peaks and antibonding peak in (211) bulk surface is unobvious and wider than that in (111) and (112). So the excited ability of electrons in (211) bulk surface is limited, which is the reason why it has the smallest surface energy.

From Fig. 3, it can be seen that the intensity of bonding peak at − 17.8 eV (labeled by ①) for microfacet is different with each other, wherein PDOS_[(110×211)]_ = 1.098 electrons/eV*atom > PDOS_[(100×211)]_ = 1.031 electrons/eV*atom > PDOS_[(112×211)]_ = 1.020 electrons/eV*atom > PDOS_[(112×112)]_ = 0.878 electrons/eV*atom > PDOS_[(111×112)]_ = 0.841 electrons/eV*atom > PDOS_[(111×211)]_ = 0.829 electrons/eV*atom, which is contrary with the trend in their cohesive energies. As bulk surface, all of the contribution to bonding electrons mainly comes from *p* orbitals and to antibonding electrons mainly comes from *d* orbitals in Mn elements^31^. At Fermi facet, the PDOSFermi [(110 × 211)] = 0.124 electrons/eV*atom < PDOSFermi [(100 × 211)] = 0.14 electrons/eV*atom < PDOSFermi [(112 × 211)] = 0.166 electrons/eV*atom < PDOSFermi [(111 × 211)] = 0.198 electrons/eV*atom < PDOSFermi [(111 × 112)] = 0.253 electrons/eV*atom < PDOSFermi [(112 × 112)] = 0.297 electrons/eV*atom, which is basically consistent with the trend of their cohesive energy. To high Miller index as (211) bulk surface and [(112 × 112)] and [(111 × 112)] microfacet, their hybridization in *p* and *d* orbital at Fermi facet (labeled by ② in Fig. 3) is obvious. But to some other bulk surface and microfacet models, such hybridization orbital is inconspicuous. As well known, the surface with high Miller index would be much more active than that with low Miller index as usual. But to (211) bulk surface, especially to [(112 × 112)] and [(111 × 112)] microfacet, their surface energies are smaller than that of other surface models. Such abnormal appearance may come from their stronger hybridization in *p* and *d* orbital.

### Deformation electronic density

**Figure 4:**
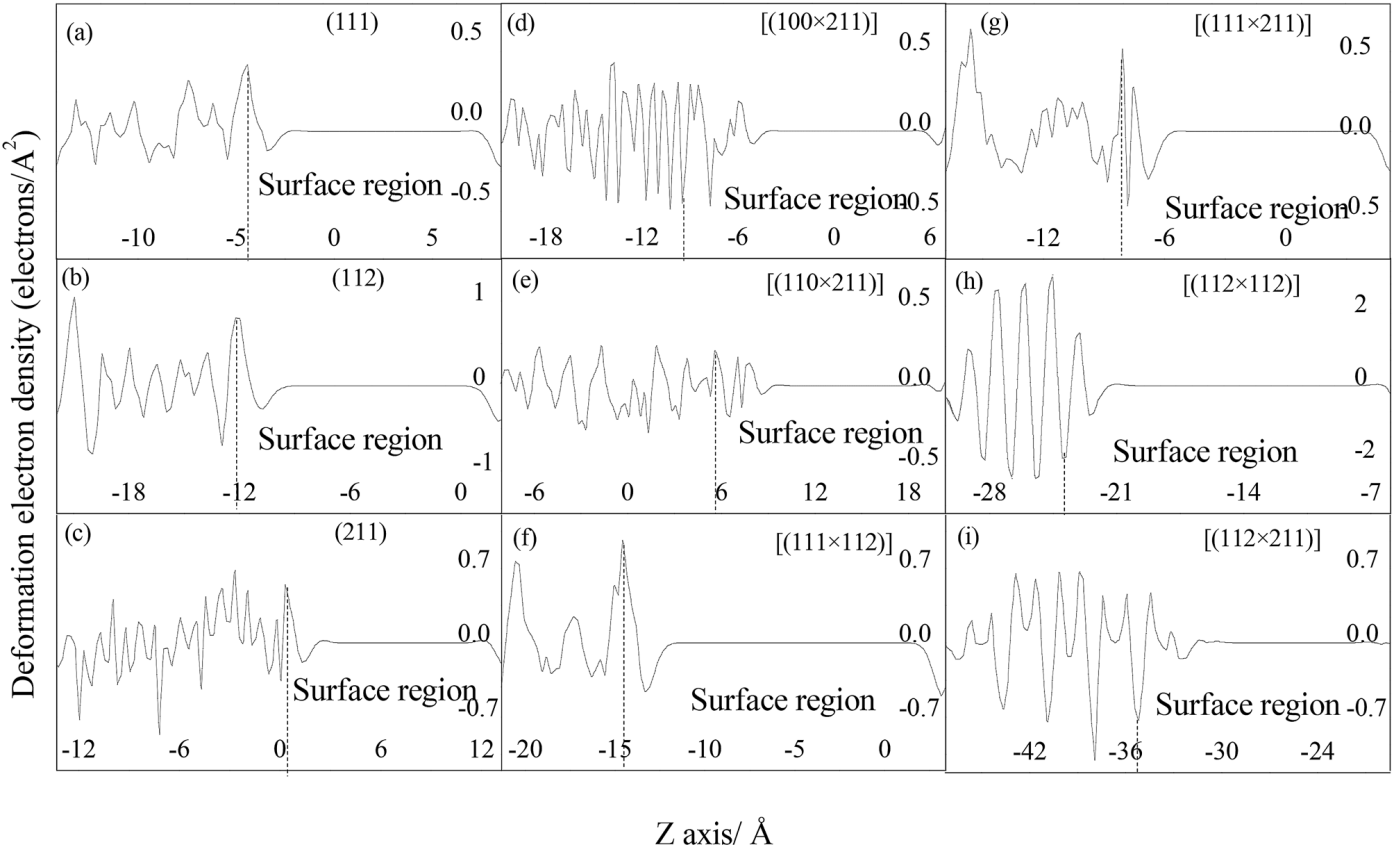
Deformation electron density of MnO_2_ bulk surface and microfacet models along Z axis.

In order to reveal their electronic bonding feature, the deformation electron density (DED) of bulk surface and microfacet were calculated as shown Fig. S2. From definition, the deformation electronic density is the total density with the density of the isolated atoms subtracted, wherein positive regions (blue region) indicate areas where bonds have formed, while negative regions (yellow region) indicate electron loss in Fig. S2. And their quantitative DED along Z axis is shown in Fig. 4, wherein positive/negative value means electrons gained/loss respectively. To investigate the contribution of electronic bonding to surface energy, their surface region was analyzed emphatically. It is found that all of the oxygen atoms is the gained electrons units and the manganese atoms is the loss electrons units, which is consistent with their results in PDOS analysis. And O element and Mn element construct covalent bond from their elliptical shape of deformation electron density as shown in Fig. S2 by arrow marked, which means they form π bonds. Their difference in surface free electrons (marked by blue and yellow color) of bulk and microfacet models may play vital role in their chemical activity as shown in Fig. S2.

However, their surface energy mainly is affected by their surface electrons and the active sites. All of the surface energies of bulk surface are smaller than that of microfacet. But the internal mechanism keeps still mysterious. Chen et al.^22^ pointed out that the larger surface energy of the microfacet comes from its large surface area. But as well known, the surface area is not contributed to the surface energy and chemical activity straightly^13^, which is only influenced by the surface electrons. From definition, the larger positive value of DED is the more powerful the covalence bond would be. And the negative value of DED means the electrons come from Mn ions. From Figs. 4 and Fig. S2, it is found that the number of positive and negative peaks in surface region of bulk surface is fewer than that of microfacet except [(112 × 211)], which means the microfacet has much more bonding location points. Compared with the character of DED in Fig. 4a–c, it is found that the height of positive peak is higher than that of negative peak for (111), (112) and (211) bulk surface in surface region. Therefore much more electrons contribute to the valance bond and leaving few free electrons along surface layer to contribute their surface energy. Compared with the character of DED in Fig. 4d–i, it is found that there exist many positive/negative peaks in surface region of microfacet, especially to [(100 × 211)], [(110 × 211)] and [(112 × 211)] in Fig. 4d,e,i. which means the microfacet has many surface bonding location points. But to [(112 × 112)] and [(111 × 112)] microfacet models, they have the fewest number of positive/negative peaks in surface region, so they have the smallest surface energies. To [(112 × 211)] (*E*_surface_ = 4.9820 Jm^−2^) microfacet, the height of negative peak is higher than that of positive peak, which means its Mn elements lose much more electrons however fewer electrons contribute the valence bond. So it has much more free electrons contributing to the surface energy. To [(100 × 211)] (*E*_surface_ = 4.1143 Jm^−2^) and [(110 × 211)] (*E*_surface_ = 4.6441 Jm^−2^), their large surface energies may come from their much more numbers of positive/negative peaks in surface region than that of [(111 × 211)], [(112 × 112)], [(111 × 112)] and bulk surface.

### Dipole moment

As well known, dipole moment can cause changes in the electric field, which can promote the separation and transfer of charge to improve the catalytic activity^32–35^. The larger the dipole moment of surface structure has, the stronger the polarity would be, which would induce much lower activation energy barrier to form chemical bonds easily^35–37^. Zhang et al.^38^ pointed out that the increase of dipole moment leaded to the increase of redox potential, which caused the increase of activity. By definition, the dipole moment can be calculated as:

3 $$ u_{{\text{i}}} = \sum\limits_{\alpha = 1}^{{\text{N}}} {q_{{{\text{i}},\alpha }} \cdot r_{{{\text{i}},\alpha }} } , $$

where *q*_i,α_ is the partial charge of atom α in particle *i* and *r*_i,α_ is the position vector of atom α in particle *i*. Then the total dipole moments are given as^33^:

4 $$ \mu_{{{\text{sum}}}} = \sqrt {\mu_{x}^{2} + \mu_{y}^{2} + \mu_{z}^{2} } . $$

In order to eliminate the effect of surface morphology, all of the total dipole moments (*μ*_sum_) were averaged by surface area (*S*). The results are shown in Table 2. It is found that the largest *μ*_sum_/*S* (0.09183 D/Å^2^) is for (112) bulk surface, which also has the largest surface energy (*E*_surface_(112) = 1.4308 Jm^−2^). And the smallest *μ*_sum_/*S* (0.08824 D/Å^2^) is for (211) bulk surface, which has the small surface energy (*E*_surface_(211) = 1.0698 Jm^−2^). And the trend in the ratio of total dipole moment divided by surface area is (*μ*_sum_/*S*) is *μ*_sum_/*S* (112) (0.09183 D/Å^2^) > *μ*_sum_/*S* (111) (0.09103 D/Å^2^) > *μ*_sum_/*S* (211) (0.08824 D/Å^2^), which is similar with their trends of surface energies. To microfacet, it is found that the largest *μ*_sum_/*S* (0.07795 D/Å^2^) is for [(112 × 211)] model, which also has the largest surface energy (*E*_surface_ [(112 × 211)] = 4.9820 Jm^−2^). And the smallest *μ*_sum_/*S* (0.05545 D/Å^2^) is for [(111 × 112)] microfacet surface, which also has the small surface energy (*E*_surface_[(111 × 112)] = 3.2259 Jm^−2^). And the trend in the ratio of total dipole moment divided by surface area (*μ*_sum_/*S*) is *μ*_sum_/*S* [(112 × 211)] (0.07795 D/Å^2^) > *μ*_sum_/*S* [(110 × 211)] (0.07720 D/Å^2^) > *μ*_sum_/*S* [(100 × 211)] (0.07517 D/Å^2^) > *μ*_sum_/*S* [(111 × 211)] (0.06326 D/Å^2^) > *μ*_sum_/*S* [(112 × 112)] (0.06106 D/Å^2^) > *μ*_sum_/*S* [(111 × 112)] (0.05545 D/Å^2^), which is consistent with their trends of surface energies. Then the dipole moments on surface slab may influence the surface activity of bulk surface or microfacet surface with defect structure. From definition, the largest dipole moment of [(112 × 211)] microfacet means it has the biggest electronic polarity. According with their deformation electronic densities in Fig. 4, the differences in polarity of (111), (112) and (211) bulk surface come from their largest different height of positive (gained electrons) and negative peaks (loss electrons). To microfacet models, their polarity may come from their number of positive (gained electrons) and negative peaks (loss electrons).

**Table 2:** Total dipole moments of MnO_2_ bulk surface and microfacet (Debye).

Models	*μ*_sum_(D)	*S*(Å^2^)	*μ*_sum_/*S*
(112)	18.46433	201.0645	0.09183
(111)	9.69884	106.5460	0.09103
(211)	10.38162	117.6548	0.08824
[(112 × 211)]	15.48775	198.6925	0.07795
[(110 × 211)]	11.53943	149.4809	0.07720
[(100 × 211)]	13.74385	182.8448	0.07517
[(111 × 211)]	13.98588	221.0766	0.06326
[(112 × 112)]	23.08529	378.0736	0.06106
[(111 × 112)]	22.35875	403.2186	0.05545

### Molecular orbital and electrostatic potential

**Figure 5:**
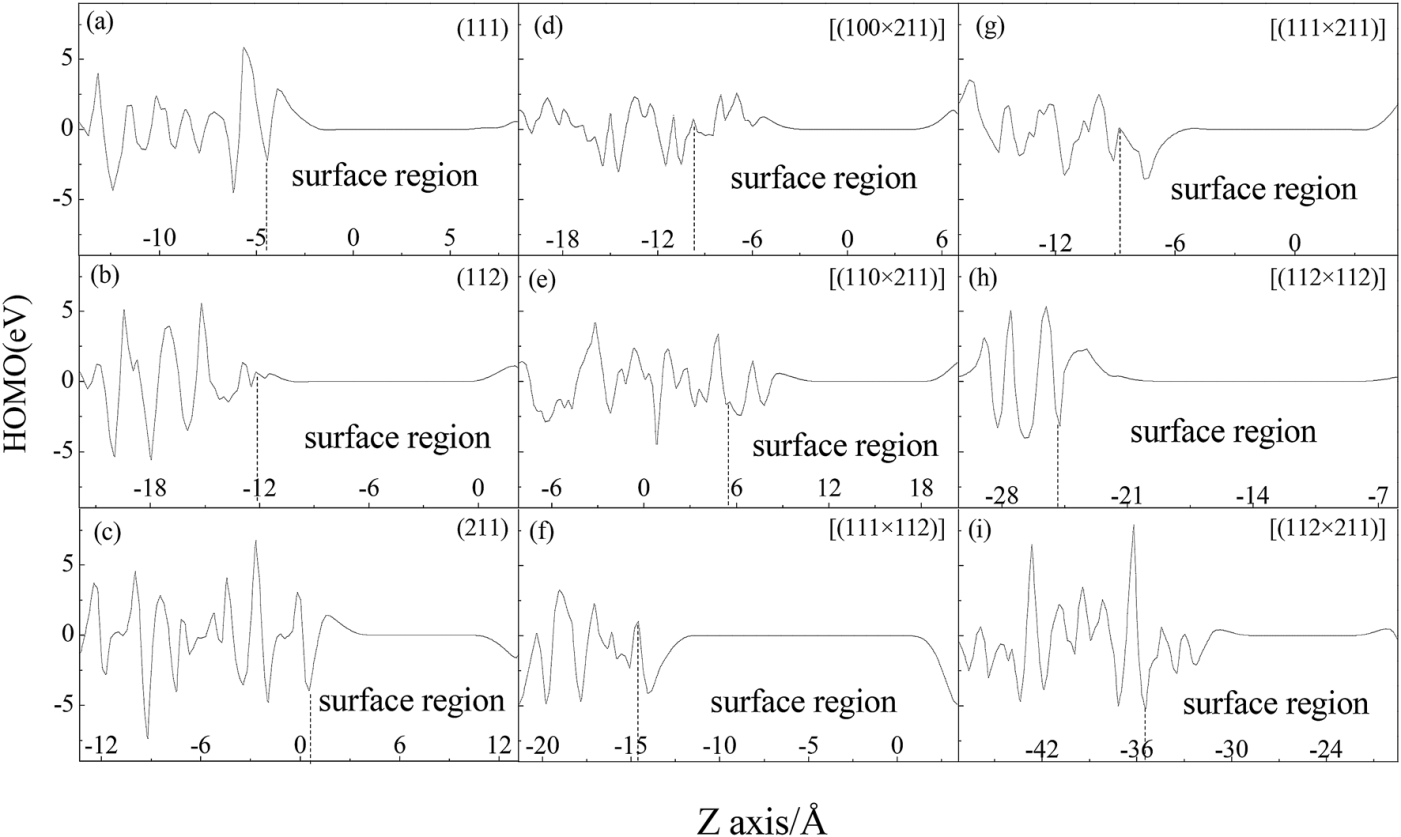
HOMO of MnO_2_ bulk surface and microfacet models.

**Figure 6:**
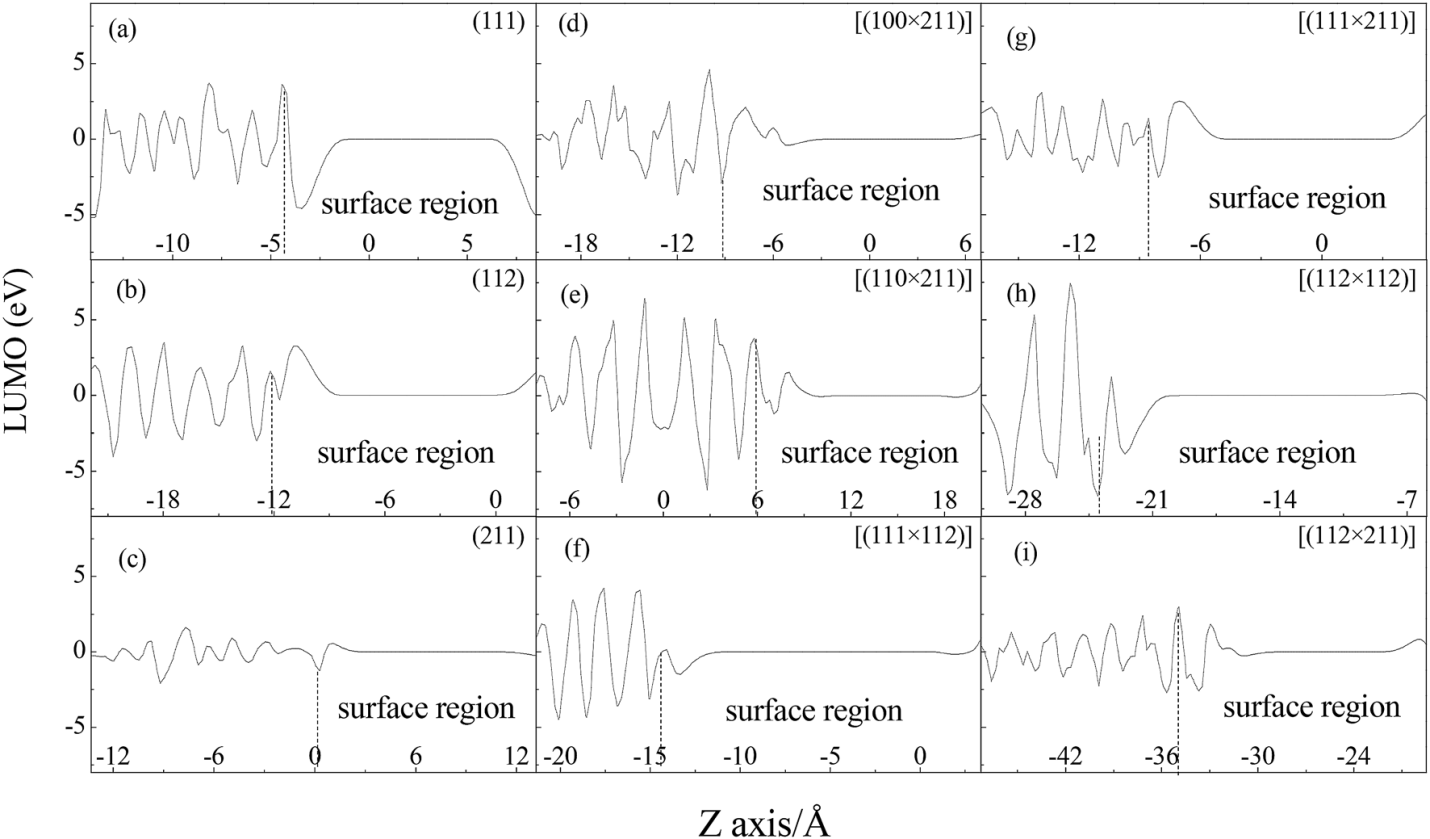
LUMO of MnO_2_ bulk surface and microfacet models.

According to frontier molecular orbital theory^34,36^, electron transfer can smoothly proceed between the highest occupied molecular orbital (HOMO) of a reducibility material and the lowest unoccupied molecular orbital (LUMO) of oxidability because these orbitals possess the same symmetry and the frontier molecular orbital of these species shares the maximum overlap. MnO_2_ is an excellent oxidant in catalytic reaction^8^. So the HOMO and LUMO of MnO_2_ bulk surface and microfacet were calculated as shown in Figs. S3 and S4. And their quantitative HOMO and LUMO along Z axis are shown in Figs. 5 and 6, wherein the positive(+)/negative(−) value represent the spin up/down respectively. To investigate the contribution of electronic orbital to surface energy, their surface region was analyzed emphatically. It is found that the number of positive and negative peaks of HOMO and LUMO in surface region of microfacet is larger than that of bulk surface in Figs. 5 and 6. So the active sites of microfacet are more than that of bulk surface with high Miller index. And the defect structure can give much more activated electrons state than bulk surface.

Otherwise, it is found that the height of positive and negative peaks of HOMO at surface region of (112) bulk surface is smaller than that of (111) and (211) bulk surface (in Fig. 5a–c). So the electrons in (112) bulk surface on HOMO have much lower energy to be activated. Furthermore, the number of positive/negative peaks in HOMO and LUMO of (112) bulk surface is equal to two, which is more than that of (111) and (211) bulk surface with one peaks. So the (112) bulk surface has lower activated energy and more motivated sites than that of (111) and (211) bulk surface, which may contribute to its largest surface energy. To microfacet, the smaller surface energies as [(112 × 112)] and [(111 × 112)] have few numbers of peaks in surface region than that of other microfacets in Figs. 5 and 6. The largest surface energy as [(112 × 211)] has many number of positive/negative HOMO peaks in surface region and the height of peaks are smaller than other microfacet (Fig. 5i). So it has many active sites and much more activated electrons to show powerful chemical activity and larges surface energy. To LUMO in Fig. 6, the number of peaks in surface region of bulk surface is also fewer than that of microfacet. For example, (111) and (211) bulk surface have one positive and negative peak, however every microfacet except [(111 × 112)] have more than two positive and negative peaks. To (112) bulk surface with largest surface energy, it has two positive peaks and one negative peak. To [(111 × 112)] with smallest surface energy, it has one positive and negative peak. The more the numbers of LUMO peaks are, the more electrons the surface layer get. So the microfacet has more powerful chemical activity than that of bulk surface.

**Figure 7:**
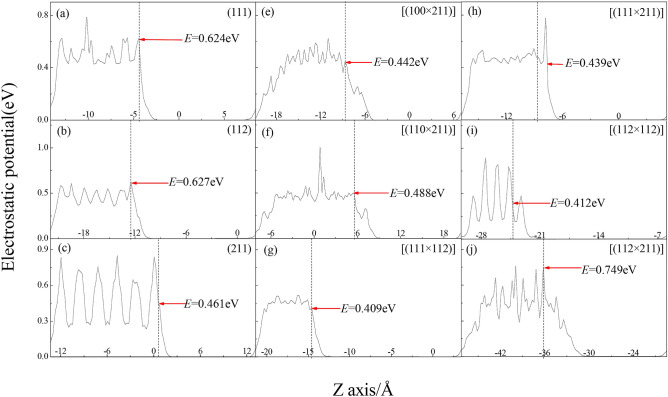
Electrostatic potential of MnO_2_ bulk surface and microfacet models.

Furthermore the electrostatic attraction contributing to adsorption and bonding has been verified by experiments and simulation analysis^36^. Thus, the electrostatic potential of bulk surface and microfacet was calculated carefully as showed in Fig. S5, wherein the electrostatic potentials range from blue to white to red means that their values range from small to large. The average electrostatic potential of a unit cell along with the Z direction is shown in Fig. 7. It is found that the trend is similar with that in HOMO and LUMO, wherein the number of electrostatic potential peaks in surface region of bulk surface is less than that of microfacet. All of the number of electrostatic potential peaks for bulk surface is equal to one and that of microfacet has more than two peaks except [(111 × 112)]. So the surface energy of bulk surface is smaller than that of microfacet, and the [(111 × 112)] has the smallest surface energy among them. To surface slab, it is found its electrostatic potential is changes along with the surface energy *E*_potential_(112) (0.627 eV) > *E*_potential_(111) (0.624 eV) > *E*_potential_(211) (0.461 eV), *E*_potential_[(112 × 211)] (0.749 eV) > *E*_potential_[(110 × 211)] (0.488 eV) > *E*_potential_[(100 × 211)] (0.442 eV) > *E*_potential_[(111 × 211)] (0.439 eV) > *E*_potential_[(112 × 112)] (0.412 eV) > *E*_potential_[(111 × 112)] (0.409 eV). So the (112) bulk surface and [(112 × 211)] have the largest power to attract bonding electrons to show largest surface energy among bulk surface and microfacet, respectively.

## Conclusion

The electronic property of defect structure and high Miller index surface in α-MnO_2_ nanorod was investigated by DFT + U method, the results show that:

For bulk surface models, the trend in surface energy as *E*_surface_(112) > *E*_surface_(111) > *E*_surface_(211) is consistent with the trend in their cohesion energy. However to microfacet models with nanostructure, the trend in surface energy as *E*_surface_[(112 × 211)] > *E*_surface_[(110 × 211)] > *E*_surface_[(100 × 211)] > *E*_surface_[(111 × 211)] > *E*_surface_[(112 × 112)] > *E*_surface_[(111 × 112)] is contrary with the trend in their cohesion energy.
(111), (112) and (211) bulk surface have one positive and negative DED peak. There exist many positive/negative DED peaks in surface region of microfacet, especially to [(100 × 211)], [(110 × 211)] and [(112 × 211)], which means the microfacet has many surface bonding location points. To [(112 × 211)] microfacet, its height of negative peak is higher than that of positive peak, which means the Mn loses much more electrons however fewer electrons contribute the valence bond.
The trend in intensity of bonding peak at − 17.3 eV for crystal and bulk surface is contrary with their cohesive energy. And the PDOS along the boundary of Fermi facet is consistent with their surface energies. To microfacet models with defect structure, it can be seen that the intensity of bonding peak at − 17.8 eV is also contrary with their cohesive energy. But the PDOS along the boundary of Fermi facet is contrary with their surface energies. Such abnormal appearance may come from their stronger hybridization in *p* and *d* orbital.
The trend in the ratio of total dipole moment to surface area is *μ*_sum_/*S* [(112 × 211)] (0.07795 D/Å^2^) > *μ*_sum_/*S* [(110 × 211)] (0.07720 D/Å^2^) > *μ*_sum_/*S* [(100 × 211)] (0.07517 D/Å^2^) > *μ*_sum_/*S* [(111 × 211)] (0.06326 D/Å^2^) > *μ*_sum_/*S* [(112 × 112)] (0.06106 D/Å^2^) > *μ*_sum_/*S* [(111 × 112)] (0.05545 D/Å^2^), which is consistent with their trend of electrostatic potential. The number of positive and negative peaks of HOMO and LUMO in surface region of microfacet is larger than that of bulk surface. So the active sites of microfacet are more than that of bulk surface.

## Supplementary Information

Supplementary Information.
