# Supplementary material for: Insight into the surface activity of defect structure in α-MnO2 nanorod: first-principles research
Source: Sci Rep. 2021 Feb 26;11:4751. doi: 10.1038/s41598-021-83861-2 (PMC7910284; doi:10.1038/s41598-021-83861-2)
Supplement: Supplementary file 1 — Supplementary Information. [file 41598_2021_83861_MOESM1_ESM.pdf]

# Insight into the surface activity of defect structure in $\alpha$-$MnO_2$ nanorod: First-principles research

Pengsen Zhao[1], Guifa Li[1,*], Haizhong Zheng[1], Shiqiang Lu[1], Ping Peng[2]

[1] *School of Material Science and Engineering, Nanchang Hangkong University, Jiangxi 330063, China;*

[2] *School of Material Science and Engineering, Hunan University, Hunan 410082, China.*

Correspondence should be addressed to Guifa Li; lgf_918@126.com

***Supplementary Material:***

**Figure S1** Sketch map of defect structures in $MnO_2$ nanorod

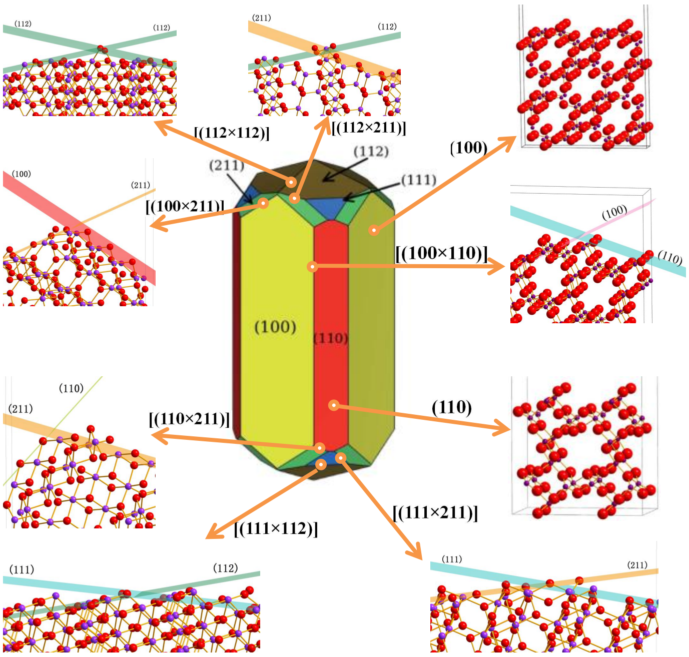

**Figure S2** Deformation electron density of $MnO_2$ bulk surface and microfacet models

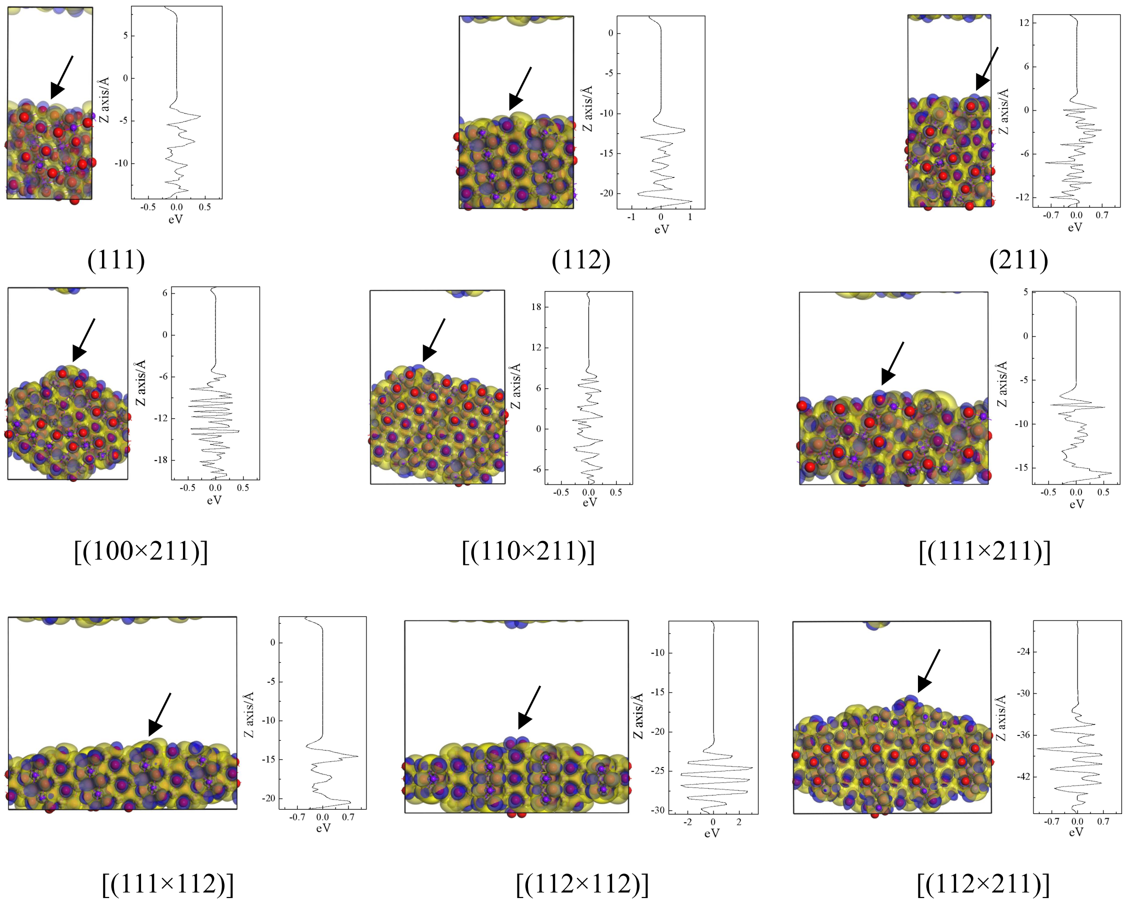

**Figure S3** HOMO of $MnO_2$ bulk surface and microfacet models

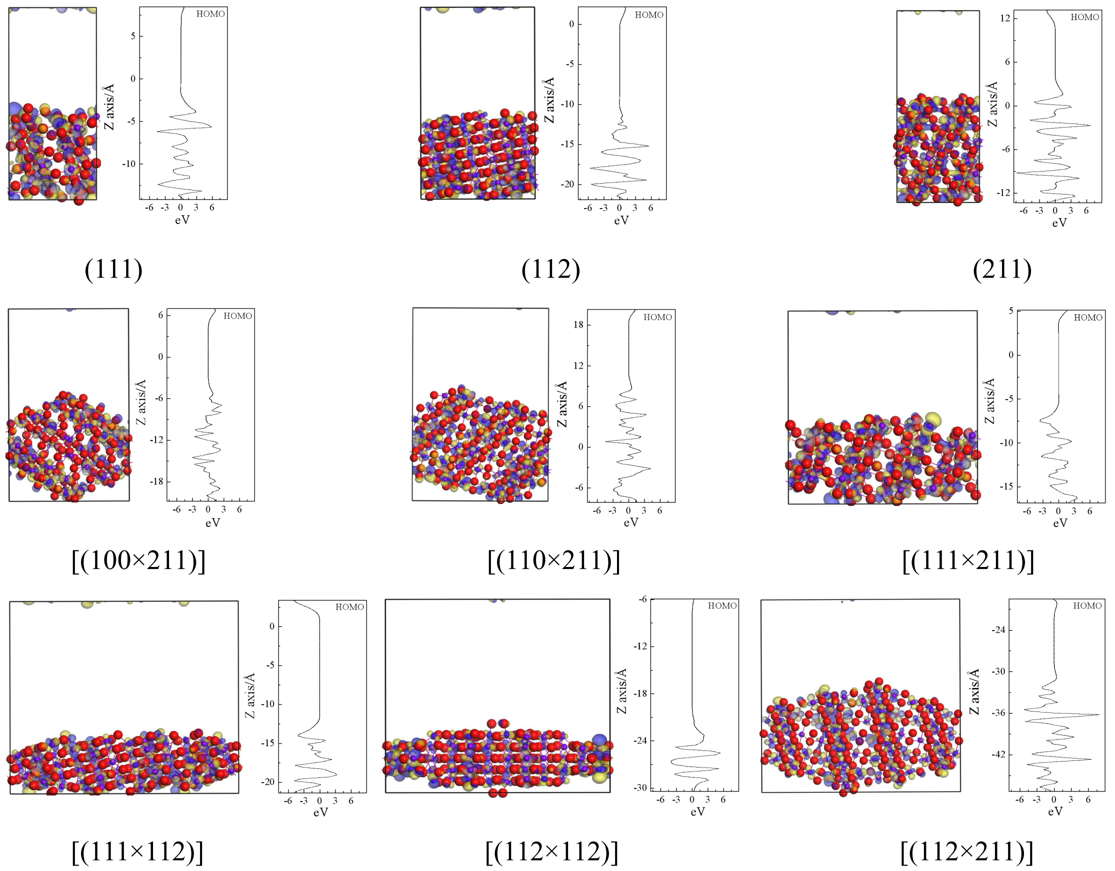

**Figure S4** LUMO of $MnO_2$ bulk surface and microfacet models

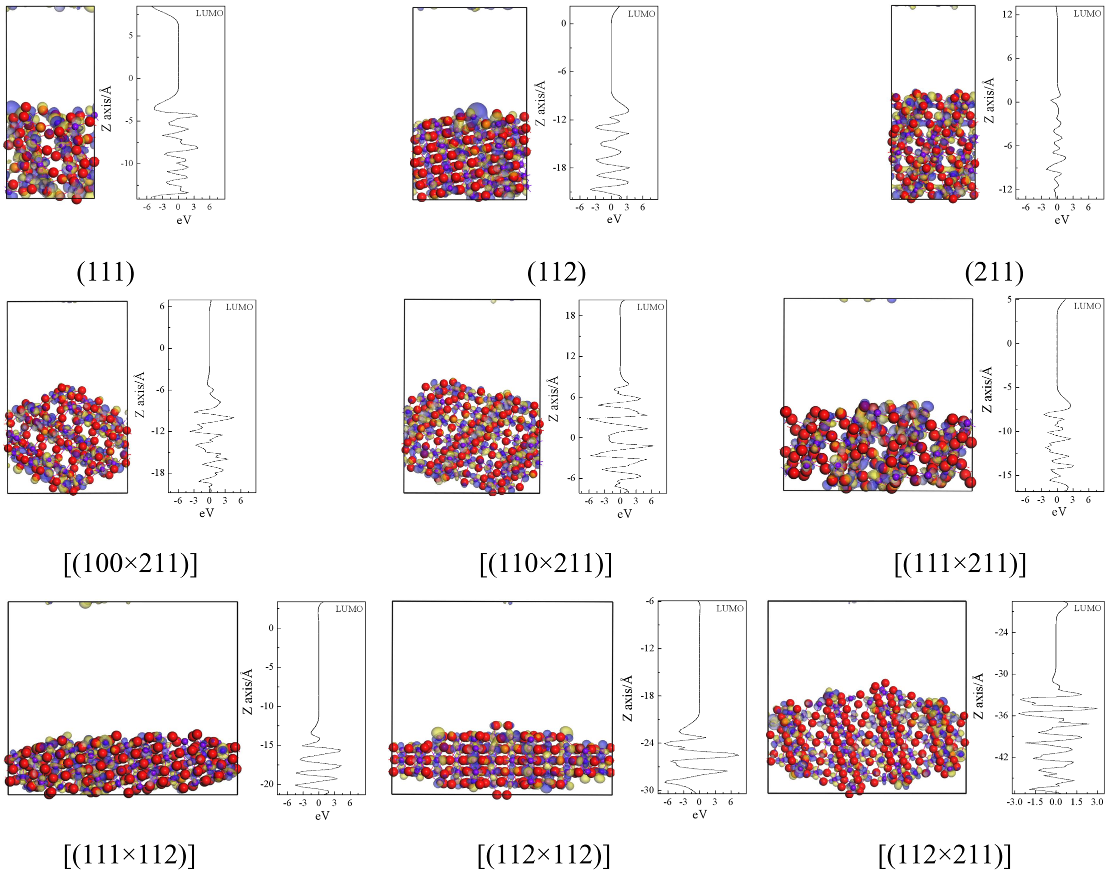

**Figure S5** Electrostatic potential of $MnO_2$ bulk surface and microfacet models

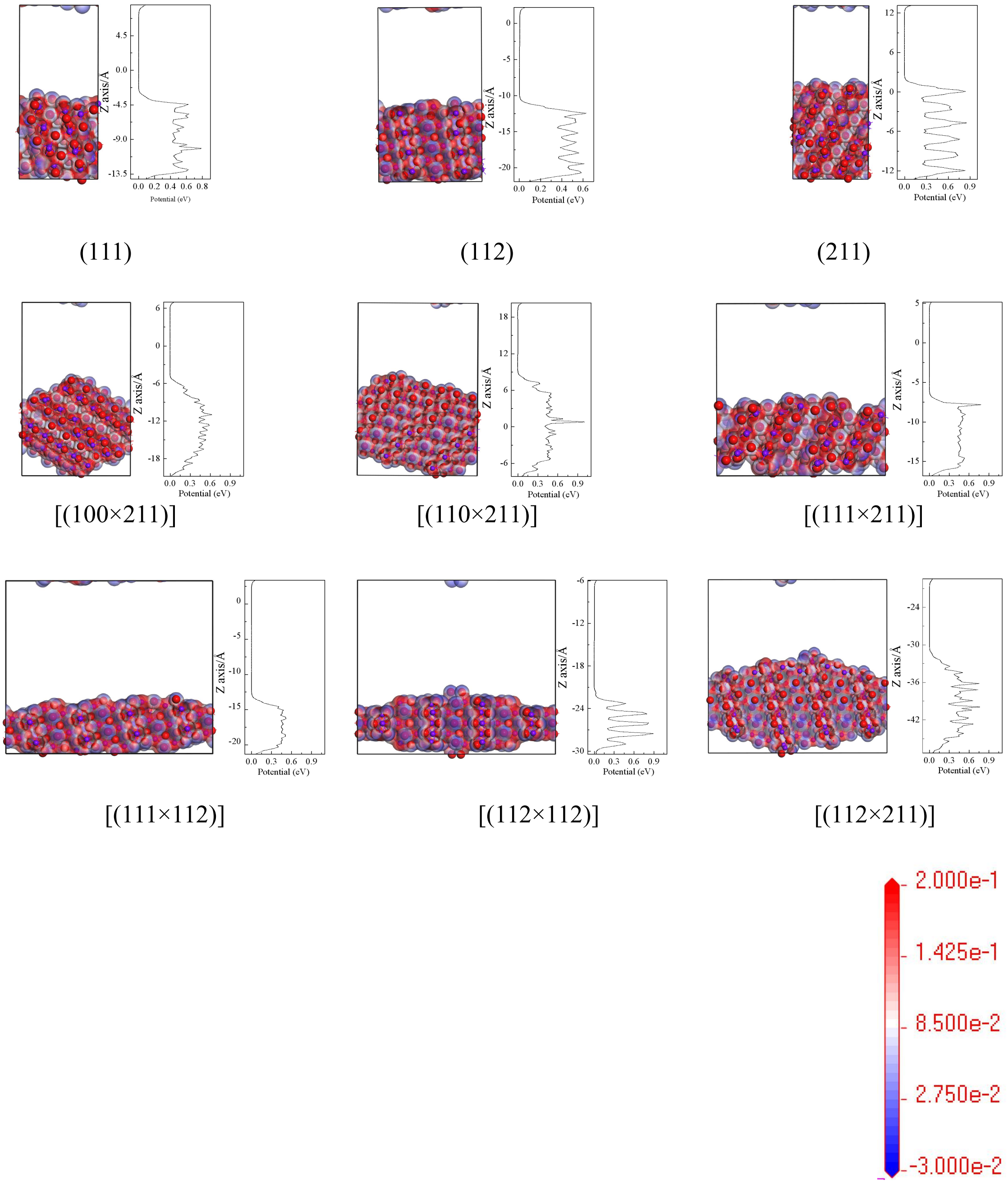

**Table S1** Lattice parameters of α-$MnO_2$ through experimental and theoretical research.

| $MnO_2$ | *a*(Å) | *b*(Å) | *c*(Å) |
|---|---|---|---|
| This work | 9.922 | 9.922 | 2.904 |
| Theo. | 9.907[13] | 9.907[13] | 2.927[13] |
| | 9.835[26] | 9.835[26] | 2.881[26] |
| Exp. | 9.750[27] | 9.750[27] | 2.861[27] |
| | 9.853[28] | 9.853[28] | 2.862[28] |
